# Research on prediction model of adolescent suicide and self-injury behavior based on machine learning algorithm

**DOI:** 10.3389/fpsyt.2024.1521025

**Published:** 2025-03-06

**Authors:** Yao Gan, Li Kuang, Xiao-Ming Xu, Ming Ai, Jing-Lan He, Wo Wang, Su Hong, Jian mei Chen, Jun Cao, Qi Zhang

**Affiliations:** ^1^ Department of Psychiatry, The First Affiliated Hospital of Chongqing Medical University, Chongqing, China; ^2^ Mental Health Center, University-Town Hospital of Chongqing Medical University, Chongqing, China

**Keywords:** machine learning algorithm, suicidal and self-injurious behavior, adolescents, risk factors, prediction model

## Abstract

**Objective:**

To explore the risk factors that affect adolescents’ suicidal and self-injurious behaviors and to construct a prediction model for adolescents’ suicidal and self-injurious behaviors based on machine learning algorithms.

**Methods:**

Stratified cluster sampling was used to select high school students in Chongqing, yielding 3,000 valid questionnaires. Based on whether students had engaged in suicide or self-injury, they were categorized into a suicide/self-injury group (n=78) and a non-suicide/self-injury group (n=2,922). Gender, age, insomnia, and mental illness data were compared between the two groups, and a logistic regression model was used to analyze independent risk factors for adolescent suicidal and self-injurious behavior. Six methods—multi-level perceptron, random forest, K-nearest neighbor, support vector machine, logistic regression, and extreme gradient boosting—were used to build predictive models. Various model indicators for suicidal and self-injurious behavior were compared across the six algorithms using a confusion matrix to identify the optimal model.

**Result:**

In the self-injury and suicide groups, the proportions of male adolescents, late adolescence, insomnia, and mental illness were significantly higher than in the non-suicide and self-injury groups (*p <*0.05). Compared with the non-suicidal self-injury group, this group also showed significantly increased scores in cognitive subscales, impulsivity, psychoticism, introversion–extroversion, neuroticism, interpersonal sensitivity, depression, anxiety, hostility, terror, and paranoia (p <0.05). These statistically significant variables were analyzed in a logistic regression model, revealing that gender, impulsivity, psychoticism, neuroticism, interpersonal sensitivity, depression, and paranoia are independent risk factors for adolescent suicide and self-injury. The logistic regression model achieved the highest sensitivity and specificity in predicting adolescent suicide and self-injury behavior (0.9948 and 0.9981, respectively). Performance of the random forest, multi-level perceptron, and extreme gradient models was acceptable, while the K-nearest neighbor algorithm and support vector machine performed poorly.

**Conclusion:**

The detection rate of suicidal and self-injurious behaviors is higher in women than in men. Adolescents displaying impulsiveness, psychoticism, neuroticism, interpersonal sensitivity, depression, and paranoia have a greater likelihood of engaging in such behaviors. The machine learning model for classifying and predicting adolescent suicide and self-injury risk effectively identifies these behaviors, enabling targeted interventions.

## 1 Introduction

Suicide and self-injury are one of the risk behaviors of adolescent health. That is, suicidal behavior disorder and non-suicidal self-injurious behavior (NSSI) can cause direct or indirect damage. They are relatively complex behavioral problems and seriously affect the mental health and safety of adolescents. At the same time, they can also lead to the loss of social resources and human costs (1, 2). Suicidal and self-injurious behaviors usually co-exist. In theory, it is easier to distinguish whether an individual who self-injures has suicidal intentions. However, in practice, it is not easy to clinically determine whether an individual has a suicidal intention when performing an injurious behavior (3, 4). Some NSSI people have suicidal intentions, especially those with poor subjective pain perception, who are more likely to have suicidal thoughts when they engage in self-injurious behavior. In addition, the data and information obtained through retrospective methods are not completely accurate, and some individuals many times deny the suicidal intention they once admitted, which may be related to shame (5–8). In recent years, the incidence of suicide and self-injury has been significantly increasing, which has sounded the alarm for us (9). Therefore, it is particularly important to explore effective methods for early identification of suicidal and self-injurious behaviors among adolescents and to provide timely intervention. Machine learning can assist in diagnosis, disease classification prediction, medical image recognition, etc. It is an important tool that can be used to assess and predict specific content in the real world (10–13). However, there are currently few studies on the application of machine learning algorithms in predicting suicidal and self-injurious behavior among adolescents. Based on the above background, this study analyzes the risk factors of adolescent suicidal and self-injurious behavior by constructing a machine learning model of suicidal and self-injurious behavior, with a view of providing prediction of suicidal and self-injurious behaviors, which offers additional possibilities.

## 2 Objects and methods

### 2.1 Research object

Stratified cluster sampling was used to select high school students in Chongqing as the research subjects. A total of 3,094 students were surveyed. After excluding questionnaires with obvious logical errors, a total of 3,000 questionnaires were recovered, with an effective response rate of 96.96%. This study was approved by the Ethics Committee of Chongqing Medical University. All research subjects were aware of the purpose and content of this study, and parents of minors were informed and signed informed consent forms.

### 2.2 Method

#### 2.2.1 Grouping

With the “Suicide Attitude and Mental Health Status Questionnaire (University Edition IV)” survey questionnaire, “Have you ever had self-injury or suicidal behavior such as drinking medication or cutting your wrist?,” the teenagers who answered “Yes” will be regarded as the self-injury suicide group, while those who answered “No” will be regarded as the non-suicide self-injury group.

#### 2.2.2 Data collection

The collection and comparative analysis of the information on gender, age, presence or absence of insomnia, and presence or absence of mental illness among adolescents were made in the suicide and self-injury groups and the non-suicide and self-injury groups.

#### 2.2.3 Model construction

Construct a classification prediction model for suicidal and self-injurious behavior based on the general information of high school students in Chongqing. The first step is to determine whether the target behavior is suicidal and self-injurious behavior, and set a two-category label, where 0=none and 1=yes. Integrate all adolescent information and construct a classification prediction model for suicidal and self-injurious behavior. Model construction is carried out with six methods, namely, multi-level perceptron, random forest, K-neighbor algorithm, support vector machine, logistic regression, and extreme gradient boosting method; comparison was made of the multi-level perceptron, random forest, K-neighbor algorithm, support vector machine, and logistic regression and various indicators of the suicide and self-injury behavior model under the six extreme gradient boosting algorithms, and the optimal model was further selected.

#### 2.2.4 The evaluation index

The confusion matrix was used to evaluate the model construction results of six methods: multi-level perceptron, random forest, K-neighbor algorithm, support vector machine, logistic regression, and extreme gradient boosting method.

### 2.3 Statistical method

The research data were analyzed by SPSS 21.0, the counting data were expressed by [n(%)], and the *x* ^2^ test was used for pairwise comparison. The measurement data that conform to the normal distribution and homogeneity of variance are expressed by (`*x* ± *s*), and the pairwise comparison passes the independent sample t-test; logistic regression model was used to analyze the independent risk factors affecting adolescents’ suicide and self-injury. The results of model construction are evaluated by the confusion matrix. *p* < 0.05 was considered as a significant difference.

## 3 Results

### 3.1 Single factor analysis of the relationship between adolescents and suicidal and self-injurious behaviors

The proportion of male adolescent, late adolescence, insomnia, and mental illness in the self-injury and suicide groups was significantly higher than that in the non-suicide and self-injury group (*p <*0.05); the cognitive subscales, impulsivity scale scores, psychoticism of adolescents in the self-injury and suicide group, introversion–extroversion, neuroticism, interpersonal sensitivity, depression, anxiety, hostility, terror, and paranoia scores were significantly increased compared with the non-suicidal self-injury group (*p <*0.05). See **Table 1**.

**Table 1:** Univariate analysis of adolescents’ relationship with suicidal and self-injurious behaviors [n (%), (`*x* ± *s)*].

Project	Self-inflicted suicide group (n=78)	Non-suicidal self-injury group (n=2,922)	*x2/t* ^_^	*p*
Gender			3.965	0.046
Male	33 (42.31)	925 (31.66)		
Female	45(57.69)	1,997 (68.34)		
Age			4.686	0.030
Late adolescence	68 (87.18)	2,242 (76.73)		
Early adulthood	10 (12.82)	680 (23.27)		
Insomnia			4.024	0.045
Have	35 (44.87)	992 (33.95)		
None	43 (55.13)	1,930 (66.05)		
Mental illness			8.355	0.004
Have	26 (33.33)	584 (19.98)		
None	52 (66.67)	2,338 (80.02)		
Cognitive subscale	51.35 ± 18.39	45.12 ± 15.37	3.562	0.001
Impulsivity scale score	48.46 ± 15.01	40.62 ± 13.87	4.562	0.000
Psychopathy	64.16 ± 12.59	54.11 ± 11.49	6.977	0.000
Introversion–extroversion	50.95 ± 10.65	48.26 ± 10.37	2.243	0.025
Neurotic	62.58 ± 11.39	52.66 ± 10.93	7.888	0.000
Interpersonal sensitivity	18.61 ± 8.23	14.51 ± 5.26	4.457	0.000
Depression	26.32 ± 10.18	19.57 ± 5.30	5.795	0.000
Anxiety	20.24 ± 8.07	14.60 ± 3.30	6.170	0.000
Hostility	12.99 ± 5.72	9.11 ± 2.51	5.937	0.000
Fear	13.10 ± 5.77	9.68 ± 2.56	5.163	0.000
Paranoid	12.55 ± 5.32	8.80 ± 2.14	6.212	0.000

### 3.2 Logistic multi-factor analysis on the relationship between adolescents and suicidal and self-injurious behaviors

Gender, age, insomnia, mental illness, cognitive subscale, impulsivity scale score, psychoticism, introversion–extroversion, and neuroticism scores were included in the logistic regression model for analysis. The results showed that gender, impulsivity scale score, psychoticism, neuroticism, sensitivity to interpersonal relationships, depression, and paranoia are all independent risk factors affecting adolescent suicide and self-injury behavior. See **Table 2**.

**Table 2:** Logistic multi-factor analysis of adolescents’ relationship with suicidal and self-injurious behaviors.

Project	*β*	*SE*	*Wald*	*p*	*OR*	95%CI
Gender	0.664	0.303	7.232	0.010	1.942	1.526–2.265
Age	0.116	0.163	3.366	0.156	1.123	0.552–1.523
Insomnia	0.23 2	0.165	8.522	0.003	1.261	0.878–1.298
Mental illness	0.11 8	0.160	4.609	0.087	1.125	0.956–1.369
Cognitive subscale	0.282	0.256	4.303	0.090	1.326	0.987–1.965
Impulsivity scale score	0.639	0.269	8.831	0.001	1.895	1.231–2.331
Psychopathy	0.940	0.352	7.587	0.008	2.561	1.958–2.989
Introversion–extroversion	0.095	0.152	4.112	0.083	1.100	0.527–1.236
Neurotic	0.67 6	0.285	8.323	0.005	1.965	1.852–3.215
Interpersonal sensitivity	0.899	0.323	8.617	0.002	2.458	1.698–4.120
Depression	0.69 9	0.269	9.660	0.000	2.011	1.859–2.963
Anxiety	0.44 6	0.362	3.403	0.154	1.562	0.958–1.653
Hostility	0.02 5	0.075	4.444	0.089	1.025	0.568–1.444
Fear	0.17 9	0.196	4.660	0.087	1.196	0.746–1.230
Paranoid	0.559	0.232	10.386	0.000	1.749	1.556–2.156

### 3.3 Analysis of different models of early warning models for adolescent suicide and self-injury behavior

**Figure 1:**
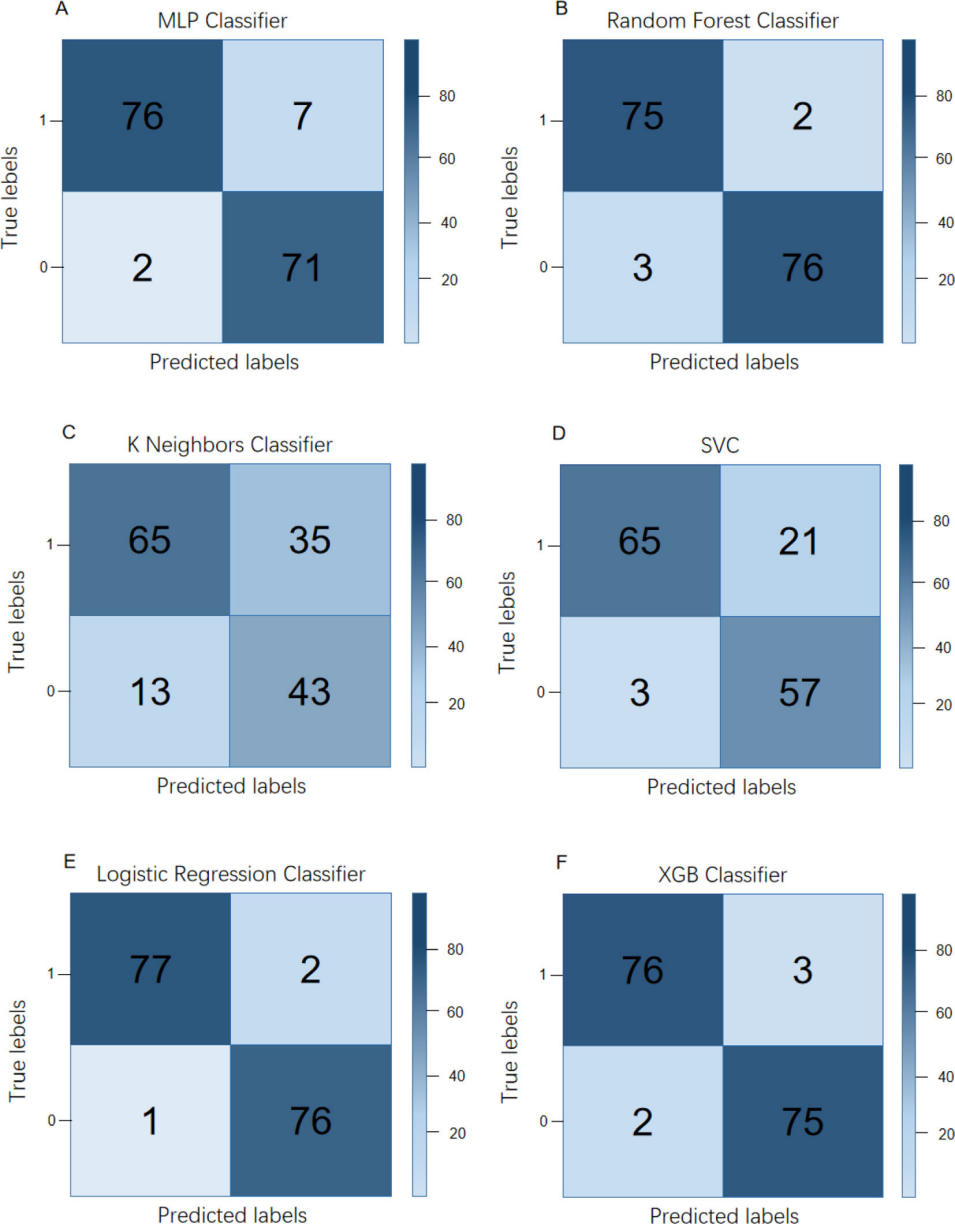
Confusion matrix of different models of adolescent suicide and self-injury behavior early warning models. **(A)** Multi-level perceptron; **(B)** random forest; **(C)** K-neighbor algorithm; **(D)** support vector machine; **(E)** logistic regression; and **(F)** extreme gradient boosting method.

The sensitivity and specificity of logistic regression in the early warning model of adolescent suicide and self-injury behavior are 0.9948 and 0.9981, respectively, which is the best performance; the performance of random forest, multi-level perceptron, and extreme gradient in the early warning model of adolescent suicide and self-injury behavior is acceptable. The K-neighbor algorithm and support vector machine performed poorly. See **Figure 1** and **Table 3**.

**Table 3:** Performance analysis of different models of early warning models for adolescent suicide and self-injury behavior.

Model type	Sensitivity	Specificity	Positive predictive value	Negative predictive value	Accuracy	AUC
Multi-level perceptron	0.9684	0.9664	0.9665	0.9683	0.9674	0.986
Random forest	0.9156	0.9826	0.9706	0.9216	0.9491	0.976
K-nearest algorithm	0.5578	0.8444	0.7775	0.6588	0.6911	0.737
Support vector machines	0.7469	0.8465	0.8283	0.7608	0.7967	0.860
Logistic regression	0.9948	0.9981	0.9980	0.9951	0.9910	1.000
Extreme gradient boosting	0.9826	0.9889	0.9887	0.9831	0.9898	0.999

## 4 Discussion

Suicide and self-injury among teenagers are a relatively complex behavioral problem that is not conducive to healthy physical and mental development. It also brings huge losses to social resources and human costs (14). The current domestic research reports on adolescent suicidal and self-injurious behaviors have a small sample size and insufficient attention, which may lead to repeated screening and missed selections, resulting in the inability to early identify adolescent suicidal and self-injurious behaviors and implement the timely and effective intervention.

The results of this study showed that 78 teenagers had suicidal and self-injurious behaviors, with a detection rate of 2.60% (78/3,000). According to the presence of suicidal and self-injury behaviors, they were divided into suicide and self-injury groups and non-suicide and self-injury groups. The outcomes showed an obvious increase in the proportion of male adolescents, late adolescence, and insomnia in the self-injury and suicide ones compared to that in the non-suicide and self-injury ones (*p* < 0.05). It had an obvious increase in the points of cognitive subscales, impulsivity scales, various dimensions of psychoticism, introversion extroversion, neuroticism, and interpersonal sensitivity, depression, anxiety, hostility, terror, and paranoia among adolescents in the self-injury and suicide ones in comparison to those in the non-suicide and self-injury ones (*p <*0.05). However, in the suicide and self-injury behavior group, the detection rate of women is higher than that of men. This may be because female adolescents are emotionally fragile and highly dependent, which leads to a higher detection rate of suicide and self-injury; in addition, compared with men, women mature psychologically earlier and are more emotionally sensitive, so they should be a key focus for preventing suicide and self-harm behavior (15, 16). Before the age of 18, adolescents are at greater risk of committing suicide and self-harm, but after the age of 18 and beyond, adolescents are more likely to engage in behaviors such as smoking and drinking (17). At the age of 18 and early adulthood, if adolescents fail to form a better identity or encounter new challenges and pressures from family and society after adulthood, they may suffer from temporary or long-term identity confusion, leading to excessive and risky behavior during the process of trial and error and, in severe cases, even deviating from social requirements (18). Research shows that family conflicts, psychological problems, interpersonal distress, and emotional problems are all important factors leading to suicide and self-harm among teenagers (19, 20). The above statistically significant variables were included in the logistic risk regression model for analysis. The results showed that gender, impulsivity scale score, psychoticism, neuroticism, interpersonal sensitivity, depression, and paranoia are independent risks that affect adolescent suicide and self-injury behavior factor. Studies such as Korczak (21) and O’Beaglaoich (22) have shown that suicidal ideation can be one of the most important proximal risk factors for suicide and can also be an independent risk factor. However, various factors for example self-esteem and social interaction can have a certain effect on suicidal ideation, which leads to a lack of specificity. The final suicide mortality rate of people with a history of suicide attempts is significantly higher than that of the general population, approximately 20–30 times; more than 50% of adolescents with a history of suicide attempts will commit suicide again in the future, and approximately one-half of those with suicidal ideation will attempt suicide within 1 year. Factors such as personality impulsivity and aggression, psychoticism, and neuroticism are inseparable from the occurrence and development of adolescent suicide and self-injury behaviors. Among them, impulsive and aggressive personalities can play a role in promoting suicide and self-injury behaviors in adolescents, especially the latter are more likely to commit suicide and self-injury. Neurotic personalities lack goals and plans, behave impulsively and blindly, and will show strong emotional reactions to the slightest stimulation, which may also be accompanied by suicide. Self-injurious behavior, in states of anxiety and depression, recent physical and mental health conditions, and the quality of intimate relationships can have an impact on adolescents, prompting them to make life and death decisions and further take action (23, 24).

Machine learning can assist in diagnosis, disease classification prediction, medical image recognition, etc. It is an important tool that can be used to judge and predict specific content in the real world. It has been widely used in the field of science and engineering and has gradually penetrated people’s daily life (25, 26). When adolescents engage in suicidal and self-injurious behaviors, large-sample studies can be used to study and further understand the social psychology related to the behavior, especially the personality and mental health of adolescents, and even key biological characteristics and risk factors, which have a crucial function in the early prediction of adolescent suicidal self-injury behavior and timely and effective intervention (27–30). In recent years, domestic and foreign scholars have used machine learning methods to construct classification and prediction models for suicide and self-injury behaviors, which have significantly improved the accuracy of predicting suicide and self-injury behaviors. For example, Wang (31) and Di (32) et al. discovered the representation of individuals with suicidal tendencies in a functional magnetic resonance imaging (fMRI) study and proposed to try to find patients with a high risk of suicidal behavior. Based on fMRI, the support vector machine recursive feature elimination method (SVM-RFE) was used to effectively identify patients with only suicidal ideation and a history of suicide attempts. The results showed that the cross-validation sensitivity was 73.10%, the specificity was 84.00%, and the accuracy was 88.20%.

At this stage, machine learning about suicide is mainly focused on the adult population, and 16–20 years old is the most common stage for mental health problems among adolescents, with approximately 75% of mental health problems occurring at this stage. From a psychological point of view, the “self-awareness” of adolescents at this stage has significantly improved, and they desire to be treated as an adult and have their personality respected. However, their independent requirements are usually ignored. Therefore, this study analyzes the risk factors of suicide and self-injury among adolescents by constructing a machine learning model of suicidal and self-injurious behavior to provide more possibilities for predicting suicidal and self-injurious behavior. The sensitivity and specificity of logistic regression in the early warning model of adolescent suicide and self-injury behavior are 0.9948 and 0.9981, respectively, which is the best performance; the performance of random forest, multi-level perceptron and extreme gradient in the early warning model of adolescent suicide and self-injury behavior is acceptable; and the K-neighbor algorithm and support vector machine performed poorly. Using machine learning methods to construct a classification prediction model for adolescent suicide and self-injury behavior is of great value for the early identification of adolescent risky behaviors. In future research, this method can also be used to analyze and predict the possible risky behaviors of other research subjects.

To sum up, the detection rate of suicidal and self-injurious behaviors in women is higher than that in men, and adolescents with impulsiveness, psychoticism, neuroticism, interpersonal sensitivity, depression, and paranoia are more likely to engage in suicidal and self-injurious behaviors. The classification and prediction model of adolescent suicide and self-injury behavior constructed using machine learning is conducive to identifying adolescent risky behaviors and providing targeted intervention for their risky behaviors. However, this study has obvious limitations. First, the obtained research content is only derived from cross-sectional data and is not analyzed in the direction of forward-looking or cohort studies, which may lead to group effects or neglect of some key points in the development process. The second point is that the time limit for suicidal and self-injurious behavior is relatively long, and this kind of behavior poses a greater threat to life. The practical value of short-term prediction is high and can effectively reduce the incidence of risk events. Third, the survey and analysis are conducted through self-reporting by adolescents. There may be concealment of risky behaviors, and there is also retrospective bias; hence, the accuracy needs to be examined.

## Data Availability

The raw data supporting the conclusions of this article will be made available by the authors, without undue reservation.
